# DNA Methylation Carries Signatures of Sublethal Effects Under Thermal Stress in Loggerhead Sea Turtles

**DOI:** 10.1111/eva.70013

**Published:** 2024-09-15

**Authors:** Eugenie C. Yen, James D. Gilbert, Alice Balard, Inês O. Afonso, Kirsten Fairweather, Débora Newlands, Artur Lopes, Sandra M. Correia, Albert Taxonera, Stephen J. Rossiter, José M. Martín‐Durán, Christophe Eizaguirre

**Affiliations:** ^1^ School of Biological and Behavioural Sciences Queen Mary University of London London UK; ^2^ Project Biodiversity, Mercado Municipal Santa Maria Ilha do Sal Cabo Verde; ^3^ Instituto do Mar (IMar), Cova d'Ínglesa Mindelo Ilha do São Vicente Cabo Verde

**Keywords:** conservation, DNA methylation, epigenetics, global warming, sea turtles, sublethal effects, thermal stress

## Abstract

To date, studies of the impacts of climate warming on individuals and populations have mostly focused on mortality and thermal tolerance. In contrast, much less is known about the consequences of sublethal effects, which are more challenging to detect, particularly in wild species with cryptic life histories. This necessitates the development of molecular tools to identify their signatures. In a split‐clutch field experiment, we relocated clutches of wild, nesting loggerhead sea turtles (*Caretta caretta*) to an in situ hatchery. Eggs were then split into two sub‐clutches and incubated under shallow or deep conditions, with those in the shallow treatment experiencing significantly higher temperatures in otherwise natural conditions. Although no difference in hatching success was observed between treatments, hatchlings from the shallow, warmer treatment had different length–mass relationships and were weaker at locomotion tests than their siblings incubated in the deep, cooler treatment. To characterise the molecular signatures of these thermal effects, we performed whole genome bisulfite sequencing on blood samples collected upon emergence. We identified 287 differentially methylated sites between hatchlings from different treatments, including on genes with neurodevelopmental, cytoskeletal, and lipid metabolism functions. Taken together, our results show that higher incubation temperatures induce sublethal effects in hatchlings, which are reflected in their DNA methylation status at identified sites. These sites could be used as biomarkers of thermal stress, especially if they are retained across life stages. Overall, this study suggests that global warming reduces hatchling fitness, which has implications for dispersal capacity and ultimately a population's adaptive potential. Conservation efforts for these endangered species and similar climate‐threatened taxa will therefore benefit from strategies for monitoring and mitigating exposure to temperatures that induce sublethal effects.

## 1 Introduction

The current pace of biodiversity loss is often referred to as the sixth mass extinction event in geological history (Barnosky et al. 2011). As global temperatures rise rapidly, conservation programmes directed at climate‐vulnerable taxa are urgently needed (IPCC 2013; Urban 2015). Mitigation strategies must integrate knowledge on how target species interact with their environment to effectively assess extinction risk and guide appropriate interventions within their unique, eco‐evolutionary contexts (Lavergne et al. 2010; Hoffmann and Sgrò 2011; Urban et al. 2016).

Species that avoid extinction typically follow two pathways (Aitken et al. 2008). They can shift in distribution (Chen et al. 2011; Lenoir and Svenning 2015), usually towards the poles (Antão et al. 2022), higher elevation (Mamantov et al. 2021) or deeper in aquatic systems (Pinsky, Selden, and Kitchel 2020). Alternatively, species may adapt in situ via genetic evolution if effective population size and reproduction rates are sufficiently high, through adaptive plastic responses, or a context‐dependent combination (Franks and Hoffmann 2012; Catullo et al. 2019). Despite those available mechanisms, responses may still be too slow to keep up with the current rate of climate change for some species (Radchuk et al. 2019). Both the ecological range‐shift and evolutionary adaptive scenarios focus on the longer‐term response to temperature increase. Yet, an immediate threat relates to the impact of sublethal physiological effects on individuals (Pörtner et al. 2006; Carlo et al. 2018; Conradie et al. 2019; Soravia et al. 2023). Characterising these effects is important in a conservation context, as negative fitness impacts on ecologically relevant functions can exacerbate extinction risk by altering population dynamics, such as reproduction and recruitment rates (Schwanz et al. 2010; Hamilton et al. 2016). Their integration will therefore strengthen predictive models and enhance the development of targeted mitigation strategies for threatened species (Chown et al. 2010; Lockley and Eizaguirre 2021).

However, sublethal effects can be difficult to measure in the wild, particularly in species with cryptic (i.e., unamenable to observation) life history stages. To overcome this challenge, conservation can benefit from molecular approaches employed in medicine, where biomarkers are frequently developed to understand and diagnose sublethal effects in patients (Silins and Högberg 2011; Sarhadi and Armengol 2022). Biomarkers are also common in ecotoxicology for characterising sublethal exposure to pesticides and pollutants (Sarkar et al. 2006; Forbes, Palmqvist, and Bach 2006; Vischetti et al. 2020). To this end, we need to identify relevant molecular signatures associated with thermal, sublethal effects in threatened species. Proteomic profiling can be altered by exposure to elevated temperatures (Abdelnour et al. 2019), but its application is hindered by labour‐intensive protocols. Gene expression levels are often associated with heat stress; however, unstable RNA can degrade rapidly post‐sampling, rendering it a complex molecule to implement as a field diagnostic marker (Desalvo et al. 2008; Lim et al. 2016; Akbarzadeh et al. 2018). An alternative is to harness the potential of DNA methylation, which is an epigenetic mechanism that dynamically regulates gene expression through the addition of a methyl group to cytosine residues (Rey et al. 2020). Methylation of promoter regions is typically thought to inhibit gene expression, although regulatory relationships throughout the genome remain unclear due to high context dependency (Jones 2012; Schübeler 2015).

Quantifying DNA methylation variation is a feasible option in the field as it can stably maintain regulatory information (Gosselt et al. 2021). Induction of methylation changes following exposure to environmental stressors such as temperature has been widely shown (Metzger and Schulte 2017; Guan et al. 2019; Sheldon et al. 2020), with links to growth, metabolism and behaviour (Guerrero‐Bosagna et al. 2020; Sepers et al. 2021). DNA methylation can also integrate environmental effects across life stages (Pértille et al. 2017; Jonsson and Jonsson 2019; Bock et al. 2022) and even across generations in some cases (Blaze and Roth 2015; Heckwolf et al. 2020). Taking these attributes together, DNA methylation profiling presents a promising approach to discovering molecular biomarkers that link thermal stress to variation in functional traits (Jeremias et al. 2020; Šrut 2021; Crossman, Barrett‐Lennard, and Frasier 2021), thus providing a practical solution for monitoring sublethal effects in wild populations.

Sea turtles are ectothermic species facing risks from warming oceans and nesting beaches due to their life cycle across both environments (Wallace et al. 2011). Vulnerability is further heightened by their temperature‐dependent sex determination system (Janzen 1994), which may feminise sex ratios to the point of demographic collapse under global temperature projections (Hawkes et al. 2009; Mitchell and Janzen 2010). Whereas much research focuses on the impact of warming on mortality and sex ratios, increasing incubation temperatures can also impose fitness costs on individuals, which could weaken the adaptive potential of the population and accelerate their decline (Eizaguirre and Baltazar‐Soares 2014; Baltazar‐Soares et al. 2020). For example, even though incubation temperatures above 34°C are often considered to be deadly for sea turtle nests (Howard, Bell, and Pike 2014; Laloë et al. 2017), hatchlings that emerge from nests incubated at low temperatures (27°C) or above 33°C experience slower growth rates than those incubated at around 30°C (Booth, Archibald‐Binge, and Limpus 2020). High temperatures can also weaken fitness‐related phenotypes, including crawling, self‐righting and swimming, thereby reducing dispersal capacity toward the ocean after hatching (e.g., Booth and Evans 2011; Fisher, Godfrey, and Owens 2014; Mueller et al. 2019; Fleming et al. 2020).

In this study, we focus on the endangered loggerhead sea turtles (*Caretta caretta)* that nest in Cabo Verde, West Africa, which is a globally important rookery threatened by coastal development, sea level rise and poaching (Taxonera et al. 2022). The population is composed of distinct genetic groups, arising from strong female philopatry down to just tens of kilometres across the ten islands of the archipelago (Stiebens et al. 2013; Baltazar‐Soares et al. 2020). Current debate exists regarding the future of this population in relation to rising incubation temperatures, with modelling studies predicting almost complete feminisation and associated demographic collapse by the century end (Laloë et al. 2014), alongside other studies arguing that this population will be resilient (Abella Perez et al. 2016). However, discussion surrounding the Cabo Verde population does not yet integrate how sublethal fitness costs on individuals will impact resilience potential, nor whether there are biomarkers to improve monitoring of such effects.

To begin addressing this challenge, we conducted a split‐clutch design experiment in an in situ hatchery, exposing clutches of wild, nesting females to different incubation temperatures under field‐relevant conditions. This was achieved by burying each sub‐clutch at either 55 cm (deep treatment) or 35 cm (shallow treatment), which exposed shallow sub‐clutches to increased temperatures that mimic warming climate projections in Cabo Verde (Laloë et al. 2014). Using a split‐clutch design enabled us to characterise developmental stress responses associated with incubation conditions, independently of the maternal background. Upon emergence, a small blood sample was collected for whole genome bisulfite sequencing to identify differentially methylated sites between treatments that could be used as biomarkers of incubation conditions. Fitness tests related to hatchling quality and locomotion capacity were also conducted and linked to methylation values at genomic sites of interest. Within the context of regional climate projections, we consider the implications of our results for the persistence of this genetically distinct and globally important loggerhead sea turtle population.

## 2 Material and Methods

### 2.1 Experimental Design

Our study focused on loggerhead sea turtles that nest on Sal Island, which is the most north‐easterly island of the Cabo Verde Archipelago in the Northeast Atlantic Ocean. There, the sea turtle nesting season runs from late June to October. The sampling site of Algodoeiro Beach (16.61773° N, −22.92882° E) covers 800 m of sandy coastline on the lower southwest of the island. On the 29th of July 2021, we relocated the clutches of 10 wild, nesting females over one night to standardise environmental conditions for all egg clutches during incubation. Immediately after oviposition, females were individually marked with a passive integrated transponder (PIT) tag on their front right flipper for identification (Stiebens et al. 2013). At the end of the natural nesting process, clutches of these 10 females (83 ± 15 (standard deviation, SD) eggs) were relocated to an in situ hatchery, where all experimental clutches were exposed to natural conditions in a protected area at the back of a nesting beach.

In the hatchery, we set up a split‐clutch design experiment to understand the sublethal effects of incubation temperature in a field setting while controlling for genetic background and maternal effects (Eizaguirre et al. 2012). Each clutch was randomly split into two sub‐clutches of equal size, hence also controlling for metabolic heating, then buried at different depths to induce different incubation temperatures experimentally. One sub‐clutch was buried at 55 cm as the ‘deep’ treatment. The other sub‐clutch was buried at 35 cm as the ‘shallow’ treatment, thereby raising the incubation temperature to mimic future conditions predicted for this population (Laloë et al. 2014). Both 35 and 55 cm lie within the natural range of egg chamber depths for wild loggerhead sea turtles in Cabo Verde, and are therefore biologically meaningful (range: 38–67 cm, Marco et al. 2018). A HOBO Pendant MX Water Temp (MX2201) temperature logger was placed at the centre of 16 out of 20 sub‐clutches and programmed to take readings every 15 min throughout the incubation period (accuracy ±0.5°C). Total incubation duration was calculated in days from the date each nest was closed to the date where most hatchlings emerged from the nest simultaneously. The mean incubation temperature was calculated across the entire incubation period. Following natural emergence, nests were excavated and the number of unhatched or dead hatchlings was counted, as well as any remaining live hatchlings that were released onto the beach for natural dispersal. Hatching success was calculated as the number of live hatchlings divided by the number of eggs per sub‐clutch.

### 2.2 Hatchling Morphometrics and Locomotion Tests

Upon emergence, all or up to 20 hatchlings per sub‐clutch (*n* = 408 in total, *n* = 198 from deep treatment, *n* = 210 from shallow treatment) were randomly selected and measured for two morphometric traits. These were the (1) notch‐to‐notch straight carapace length (SCL, in millimetres, mm) and (2) mass (in grams, g). SCL was recorded as the mean of three measurements per hatchling using a digital calliper (±0.01 mm), ensuring all measurements fell within a range of 0.5 mm. Mass was measured once per hatchling using a digital scale (±0.1 g).

In addition, two fitness tests of locomotion capacity were conducted: (1) run time and (2) self‐righting time. These traits are important components of predator and obstacle avoidance, which are both required to successfully reach the ocean after emerging from the nests (Scott et al. 2014; Lockley et al. 2020; Martins et al. 2020). We measured run time as the time taken for a hatchling to crawl along a 0.5 m runway of flat sand between two wooden pieces, with a dull red light at the end of it. This trial was repeated twice per hatchling. If a hatchling did not attempt to crawl at all, it was considered to have failed the trial. The mean run time (in seconds, s) was then calculated across the successful trials per hatchling. Self‐righting time was measured by placing a hatchling on its back on an area of flat sand and timing how long it took to right itself. This trial was repeated three times per hatchling. If a hatchling took longer than 1 min to self‐right, it was considered to have failed the trial. We then measured the mean self‐righting time (s) using the successful trials per hatchling. All fitness tests were conducted between the 16 and 25th September 2021, during which the mean ambient temperature at night was 26.3 ± 0.48 (SD)°C.

### 2.3 Clutch‐ and Hatchling‐Level Phenotype Analyses

**FIGURE 1:**
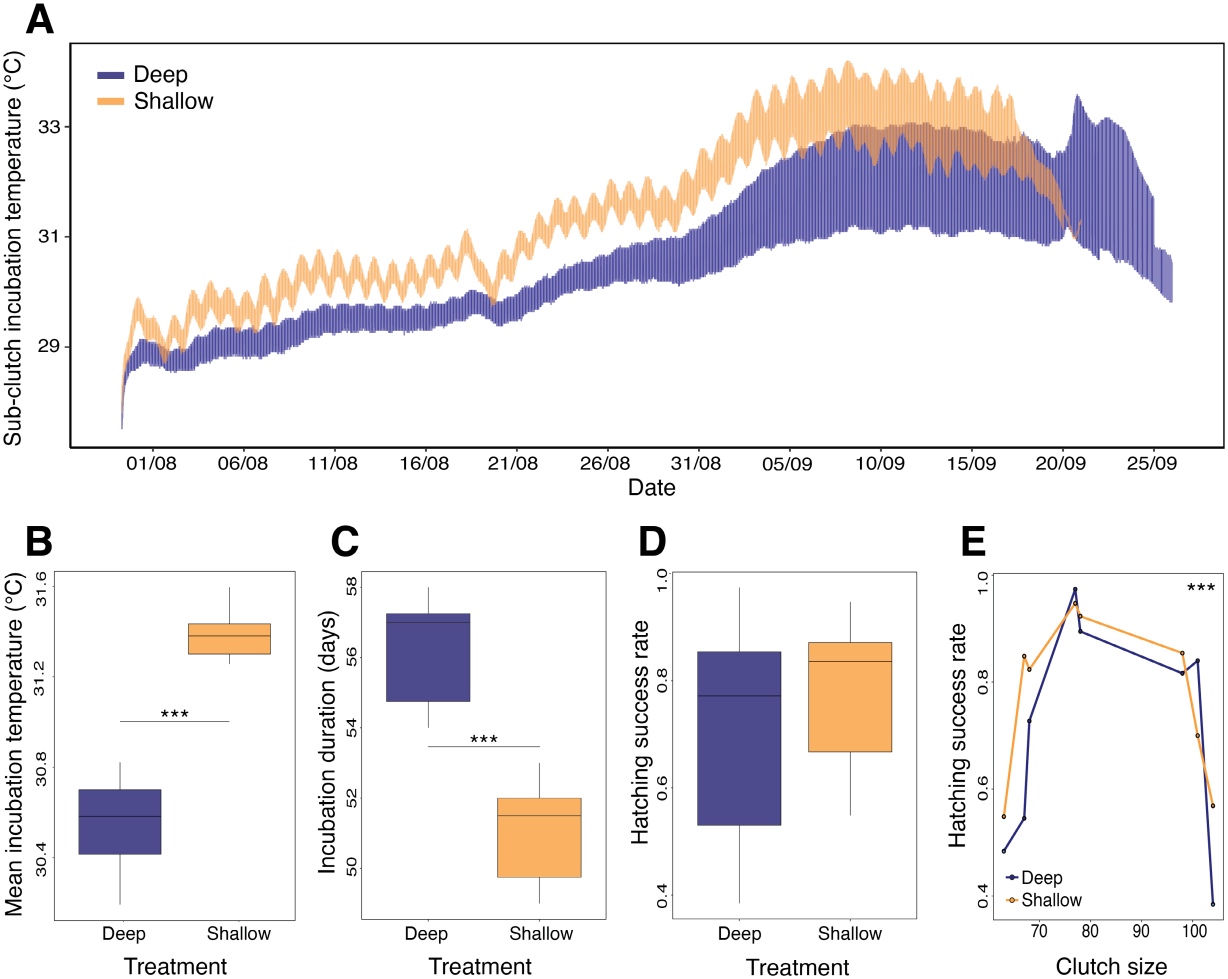
Clutch‐level incubation temperature and phenotypes. Deep sub‐clutches are shown in blue and shallow sub‐clutches in orange. ‘***’ indicates a significance level of *p* < 0.001. (A) Temperature readings (°C) overlapped across all temperature loggers over the full incubation period (*n* = 16). Shallow sub‐clutches reached higher temperatures than deep sub‐clutches throughout the incubation period, with daily fluctuations visible. (B) Sub‐clutches incubated at the shallow treatment experienced higher mean incubation temperatures (°C) than the deep treatment. (C) Incubation duration (days) was longer in sub‐clutches incubated in the deep treatment than in the shallow treatment. (D) No significant difference in hatching success rate was found between sub‐clutches incubated in deep and shallow treatments. (E) Large and small total clutch sizes exhibited lower hatching success rates compared to intermediate clutch sizes.

All statistical analyses on phenotypic traits were carried out in RStudio v.4.2.2 (R Core Team 2021), with full model formulations and outputs provided in Table S2. Firstly, we used linear models to investigate associations at the clutch‐level. We tested whether the mean sub‐clutch incubation temperature was different between the depth treatments (Table S2A). We also tested if sub‐clutch incubation duration was associated with depth treatment, mean incubation temperature and their interaction (Table S2B). To account for the strong correlation between depth treatment and incubation temperature, we calculated the residuals of a linear model between those variables and used them to replace mean sub‐clutch incubation temperature in subsequent models looking for correlates of fitness‐related traits. Hereafter, this variable is referred to as ‘sub‐clutch incubation temperature residuals’. This was necessary as more parameters than temperature may be impacted by depth treatment, such as humidity, oxygen concentration or the microbiome. We tested if sub‐clutch hatching success rate correlated with depth treatment, sub‐clutch incubation temperature residuals, full clutch size and its quadratic term, as well as all two‐way interactions with depth treatment (Table S2C). Full clutch size was included in the model because it is qualitatively identical to sub‐clutch size but is more biologically relevant to hatching success as it reflects maternal effects, such as investment strategy. Clutch size was also included to account for metabolic heating and other nest microclimate alterations due to egg number. The quadratic term of clutch size was included due to the quadratic relationship found between hatching success and clutch size (Figure 1E). Two‐way interactions between depth treatment and clutch size variables were included to capture context‐dependent effects between incubation condition and maternal effects. The interaction between depth treatment and ‘sub‐clutch incubation temperature residuals’ served to capture some of the complexity induced by depth manipulation in field conditions, outside of temperature differences. We performed backward stepwise selection with the ‘stepAIC’ function of the R package MASS v.7.3.60 to retain relevant variables of the model (Venables and Ripley 2002).

Secondly, we focused on detecting correlations with hatchling fitness traits at the individual‐level. We used a series of linear mixed‐effects models in the R package lme4 v.1.1‐33 (Bates et al. 2014), with maternal ID included as a random effect to account for genetic and maternal effects. We investigated correlates of hatchling size (SCL), considering depth treatment, sub‐clutch incubation temperature residuals, sub‐clutch size and all two‐way interactions with depth treatment (Table S2D). We also performed the same model with mass included, to investigate the size–mass relationship of hatchlings (Table S2E). Sub‐clutch size was included in models to represent the effect of egg number (i.e., metabolic heating) and nest microclimate. Mean run and self‐righting times were log_10_ + 1 transformed and tested for correlations with depth treatment, sub‐clutch incubation temperature residuals, sub‐clutch size and all two‐way interactions with depth treatment, with SCL considered as a covariable (Table S2F–G). All models were backward stepwise selected using the ‘step’ function of the R package lmerTest (Kuznetsova, Brockhoff, and Christensen 2017). All figures were made using the R package ggplot2 v.3.4.2 (Wickham 2016).

### 2.4 Sample Collection for Molecular Work

Immediately after morphometric measurements and completing fitness tests, 100 μL of blood was sampled from the dorsal cervical sinus of each hatchling, using a 26‐gauge needle and 1 mL syringe (Wibbels et al. 1998). Collected blood samples were stored in lithium‐heparin‐coated tubes and refrigerated for up to 12 h. Samples were then centrifuged for 1 min at 3000 rpm to separate plasma and blood cells. These samples were stored at −18°C until the end of the field season, then at −80°C following transport to Queen Mary University of London (London, UK).

### 2.5 DNA Extraction and Sequencing

Blood cell samples from a subset of 40 hatchlings (20 hatchlings per deep and shallow treatment) were selected for whole genome bisulfite sequencing (WGBS), with two hatchlings sequenced per sub‐clutch. Within each sub‐clutch, a hatchling was chosen for sequencing if it had not failed any fitness tests, with priority given to hatchlings that were sampled earlier in the sub‐clutch to reduce effects of waiting time on fitness test performance. We made the conservative decision to avoid sequencing hatchlings that failed tests, as we wanted to avoid sequencing hatchlings with outlier phenotypes that would mask the subtler sublethal effects we are interested in capturing. Genomic DNA was extracted from the 40 blood cell samples using a QIAGEN Blood and Tissue Kit (Qiagen, Germany) following the manufacturer's protocol. DNBseq whole genome bisulfite libraries were constructed and sequenced with 100 base pair (bp) paired end reads on an MGI DNBSEQ platform (BGI, Hong Kong). This generated a mean of 134,492,469 ± 3,641,276 (SD) reads per sample (Table S1).

### 2.6 Read Trimming and Methylation Calling

All bioinformatic steps were conducted on the Apocrita High Performance Computing Cluster (King, Butcher, and Zalewski 2017). WGBS reads were trimmed for residual adapters and filtered for a mean Phred score higher than Q20 using cutadapt v.2.10 (Martin 2011). Read quality control was conducted pre‐ and post‐trimming with FastQC v.0.11.9 (Andrews 2010). Using Bismark v.0.22.1 with default options (Krueger and Andrews 2011), trimmed reads were aligned against our novel, chromosomal‐scale reference genome assembly for a loggerhead sea turtle from Sal Island, Cabo Verde (Yen et al. 2024). This gave a mean mapping efficiency of 78.5 ± 3.4 (SD)% per sample (Table S1). Alignments were deduplicated with Bismark in paired end mode, followed by merging and sorting with samtools v.1.9. (Li et al. 2009). Methylation calling was then performed in Bismark. Percentage methylation totalled across CHG and CHH sites was calculated to obtain an estimate of bisulfite conversion efficiency (Laine et al. 2023). This gave a mean of 99.993 ± 0.002 (SD)%, supporting high conversion efficiency (Table S1). We focused on methylation patterns at CpG sites for our study, since this is the major methylation context in vertebrates (Klughammer et al. 2023). To improve coverage and minimise pseudo‐replication, we further de‐stranded adjacent cytosines per CpG site using the ‘merge_CpG.py’ script (Cristofari 2022), as methylation occurs symmetrically at CpG sites in vertebrates (Klughammer et al. 2023). On average, this resulted in 24,947,580 ± 825,959 (SD) CpG sites per sample, with a de‐stranded coverage of 8.56 ± 0.93 (SD) X (Table S1).

Methylation calls at CpG sites were further processed in RStudio v.4.2.2 (R Core Team 2021) with the methylKit package v.1.24.0 (Akalin et al. 2012). CpG sites were excluded if they had a coverage lower than 5× or if they were within the 99.9th percentile to account for possible PCR bias (Wreczycka et al. 2017). Coverage was then normalised between samples using methylKit's ‘normalizeCoverage’ function. Following coverage filtering, we implemented a stringent filter that only passed CpG sites covered in all individuals (*n* = 40). This is because we are interested in identifying robust sites that could be developed as universal biomarkers for this population. As a final filtering step, CpG sites that were potential C‐to‐T single nucleotide polymorphisms (SNPs) were removed because these can be misidentified as non‐methylated Cs in bisulfite‐treated DNA and subsequently bias differential methylation estimates (Wreczycka et al. 2017). To obtain a list of C‐to‐T SNP sites to remove, the Revelio algorithm was used to mask bases generated by the bisulfite conversion process that may be incorrectly interpreted as SNPs (Nunn et al. 2022), and then the GATK pipeline v.4.2.6.1 (McKenna et al. 2010) was used to call SNPs (Text S1 for full methods). We removed all CpG sites sharing genomic coordinates with identified C‐to‐T SNPs, leaving 2,733,573 CpG sites (99.77%) for downstream analyses.

### 2.7 Global Methylation Analyses

To characterise changes in the entire DNA methylome in response to incubation treatment, we compared global methylation patterns between hatchlings from the deep and shallow treatments at 2,733,573 CpG sites. Using methylKit's ‘percMethylation’ function, matrices of percentage methylation values per individual at each CpG site were generated. Methylation level per individual was obtained by counting the number of sites that exhibited (1) non‐zero methylation and (2) a methylation percentage greater than 70% (Sagonas et al. 2020). Using the R package lme4 v.1.1.35.3 (Bates et al. 2014), correlations between methylation count and treatment were tested using linear mixed‐effect models with maternal ID as a random effect (Table S2H,I). We further performed cluster analyses of global methylation patterns, with sites of low variation across individuals (SD < 0.3) excluded since these are non‐informative for clustering (Sagonas et al. 2020). Hierarchical clustering was performed using methylKit's ‘clusterSamples’ function with the Ward agglomeration method and correlation distance method. Non‐metric multidimensional scaling plots (NMDS) were computed using the Bray–Curtis dissimilarity matrix with *k* = 6 dimensions and a maximum of 1000 iterations through the ‘metaMDS’ function of the R package vegan v.2.6.4 (Oksanen et al. 2022). To assess the contribution of treatment group and maternal ID to methylation variance, permutational multivariate analysis of variance (PERMANOVA) was conducted using vegan's ‘adonis2’ function with 999 permutations.

### 2.8 Identification of DMS Between Incubation Treatments

To identify differentially methylated sites (DMS) between hatchlings incubated in the deep and shallow treatments, we used the R package PQLseq v.1.2.1 (Sun et al. 2019). PQLseq runs a binomial mixed effects model to test for differential methylation between treatments per site while accounting for genetic covariance by considering a relatedness matrix between individuals as a random effect (Lea, Tung, and Zhou 2015; Sun et al. 2019). Our input kinship matrix was constructed by setting the relatedness of siblings from the same clutch to *r* = 0.5 and hatchlings from different clutches to *r* = 0, as done in von Holdt et al. (2023). After running PQLseq with default options, non‐converging sites were removed, and then the sliding linear model method (SLIM) was applied for multiple testing correction (Wang, Tuominen, and Tsai 2011). The methylation difference at each CpG site was also calculated as the mean difference between hatchlings from the deep treatment minus the shallow treatment, weighted by read coverage (Akalin et al. 2012). We considered a CpG site to be differentially methylated if it had a mean methylation difference greater than 10% between hatchlings from the two treatments and a *q*‐value less than 0.05. We further performed hierarchical clustering, NMDS and PERMANOVA analyses with the subset of identified DMS sites only, using the same parameters as described previously for global methylation analyses.

### 2.9 Annotation of Genomic Location and Genes Associated With DMS

To annotate the type of genomic region upon which CpG sites reside, we used the R packages genomation v.1.30.0 (Akalin et al. 2015) and GenomicRanges v.1.50.2 (Lawrence et al. 2013) alongside our reference genome annotation (Yen et al. 2024). Promoter regions were defined as 1500 bp upstream and up to 500 bp downstream of a transcriptional start site (TSS, Heckwolf et al. 2020). All CpG sites were assigned to one of four genomic categories using genomation's ‘annotateWithGeneParts’ function, in the following order of precedence when features overlapped: promoter, exon, intron or intergenic region. A chi‐squared test was used to evaluate whether DMS were differently distributed across genomic region types compared to all CpG sites. To attach functional gene information to our DMS, those in genic regions (i.e., located on a promoter, intron or exon) were associated with a gene using the ‘findOverlaps’ function of GenomicRanges. DMS in intergenic regions were associated to a gene using genomation's ‘getAssociationWithTSS’ function if they were less than 10 kb away from the nearest TSS (Heckwolf et al. 2020).

### 2.10 Correlating Methylation Value at DMS of Interest Against Fitness‐Related Phenotypes

To investigate the link between hatchling phenotype and molecular responses to incubation conditions, we tested for correlations between hatchling fitness‐related traits and methylation values for a subset of DMS. DMS were selected for testing if the mean methylation difference between hatchlings from deep and shallow treatments was greater than 20%, resulting in 29 DMS of interest. We performed a series of independent linear mixed effect models testing for the interaction between methylation value at DMS of interest with depth treatment, for hatchling SCL, mass, log_10_ + 1 transformed mean run and self‐righting times (Table S2J–M). The interaction was investigated to capture the environmental context‐dependency of methylation changes. Maternal ID was set as a random factor. *p*‐values were corrected for multiple testing (29 DMS × 4 fitness traits) with the Benjamini‐Yekutieli method for a significance threshold of 0.05, giving an FDR (false discovery rate) adjusted *p*‐value threshold of 0.0083 (Benjamini and Yekutieli 2001).

### 2.11 Functional Enrichment Analyses

For all genes associated with DMS that had attached Gene Ontology (GO) terms in the loggerhead sea turtle genome annotation (*n* = 94 genes, Yen et al. 2024), we performed a conditional hypergeometric GO term enrichment analysis using the R packages GOstats v.64.0 (Falcon and Gentleman 2007) and GSEABase v.1.60.0 (Morgan, Falcon, and Gentleman 2023). The gene sub‐universes were assigned as genes associated with DMS, split into hyper‐methylated (i.e., higher methylation values in deep‐incubated hatchlings, *n* = 110) and hypo‐methylated sites (i.e., higher methylation values in shallow‐incubated hatchlings, *n* = 137). These were compared against the gene universe, which was set as all genes associated with CpG sites present in all individuals with GO terms available (*n* = 13,681 genes). Overrepresented biological processes, molecular functions and cellular components were then identified using the FDR method with a threshold of 0.05 for multiple testing correction.

## 3 Results

### 3.1 Clutch‐Level Phenotypes

The incubation period lasted 53.6 days on average (minimum 49 days, maximum 58 days), over which the mean incubation temperature ranged from 30.2°C to 31.6°C (Figure 1A). Incubation duration and mean incubation temperature per sub‐clutch were strongly negatively correlated (*R* ^2^ = 0.88, *F*_1,14_ = 115.49, *p* < 0.001, Figure S1A), as expected for a well‐known effect in this species. To confirm that depth treatment was associated with different temperatures, we compared the mean incubation temperature between sub‐clutches buried at deep and shallow depths with temperature loggers (16 sub‐clutches in total, 8 sub‐clutches per depth treatment). Shallow sub‐clutches were warmer than deep sub‐clutches (*F*_1,14_ = 100.69, *p* < 0.001) by a mean of 0.83°C (Figure 1B). Shallow sub‐clutches also experienced higher maximum temperatures (*T*_deep_ = 32.64°C ± 0.67°C, *T*_shallow_ = 33.77°C ± 0.40°C, Model statistics: *F*_1,14_ = 16.64, *p* = 0.001) and increased temperature fluctuations (mean standard deviation, SD_deep_ = 1.24°C; SD_shallow_ = 1.42°C, *F* = 2.91, *p* = 0.11) compared to deep sub‐clutches. Hence, the shallow treatment matches predicted conditions of increased temperatures and variability before the end of the century (Laloë et al. 2014).

Increased temperatures translated into faster egg development and shorter incubation duration for the shallow sub‐clutches (*F*_1,12_ = 17.33, *p* < 0.001, Figure 1C). Shallow sub‐clutches hatched on average 5 days earlier than deep sub‐clutches (shallow sub‐clutches = 50.7 ± 1.67 days, deep sub‐clutches = 56.2 ± 1.51 days). Hatchlings emerged from all 40 sub‐clutches, with a mean of 34 ± 9 (SD) hatchlings per sub‐clutch. There was no difference in hatching success rate of sub‐clutches based on depth treatment (*F*_1,12_ = 2.101, *p* = 0.173, Figure 1D). Interestingly, we instead found that hatching success was a trait correlated with the full clutch size, with small and large clutch sizes exhibiting a lower hatching success rate than intermediate sizes (quadratic term for clutch size, *F*_1,12_ = 41.83, *p* < 0.001, Figure 1E). Noteworthy, we could detect the effects of metabolic heat, as demonstrated by the positive correlation between incubation temperature and sub‐clutch size, independently of the depth treatment (*F*_1,12_ = 23.036, *p* < 0.001; Treatment × Sub‐clutch size: *F*_1,12_ = 1.3166, *p* = 0.273, Figure S1B).

### 3.2 Hatchling‐Level Morphometric and Locomotion Phenotypes

**FIGURE 2:**
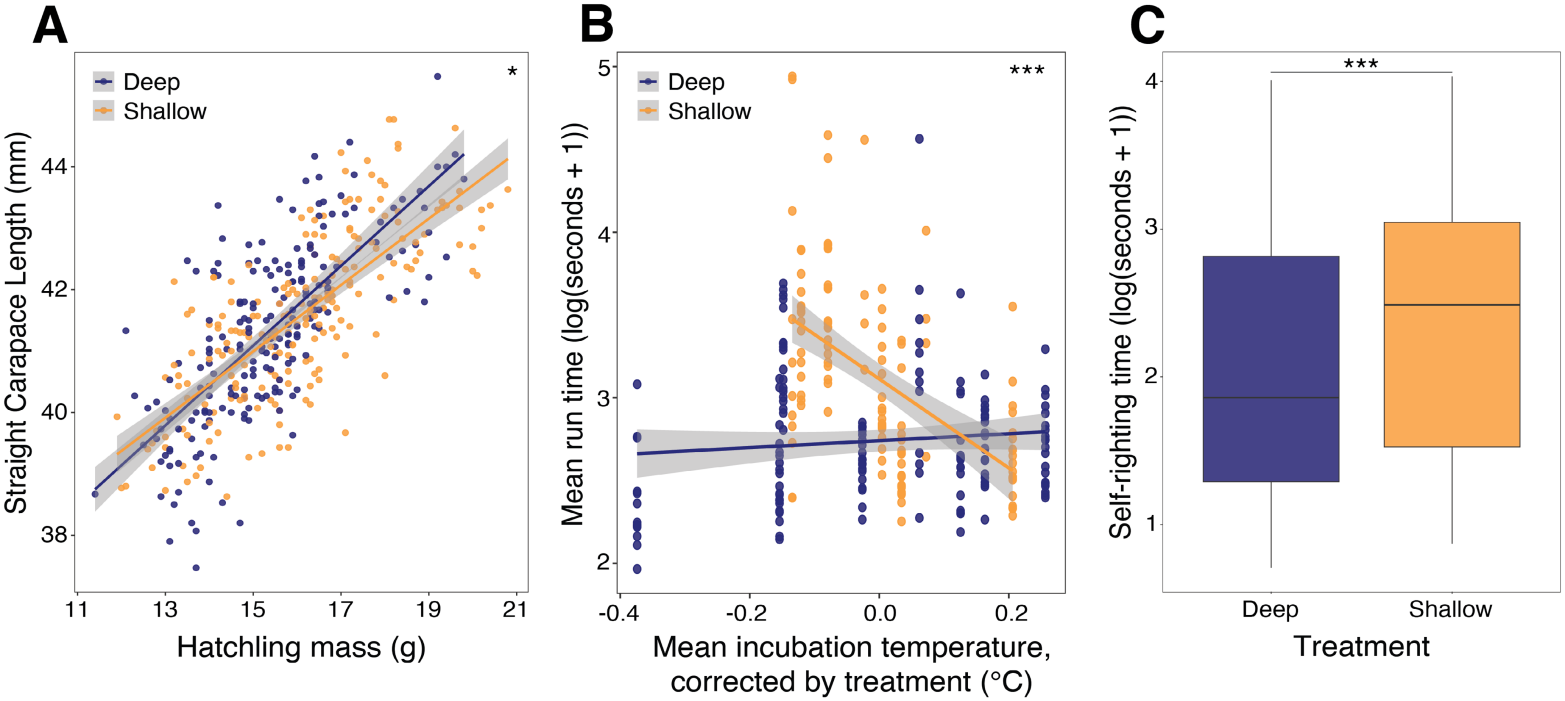
Individual‐level hatchling phenotypes. Hatchlings emerging from deep sub‐clutches are shown in blue and those from shallow sub‐clutches in orange. ‘*’ indicates a significance level of *p* < 0.05 and ‘***’ indicates a significance level of *p* < 0.001. (A) Hatchling straight carapace length (SCL, mm) is positively correlated with mass (g), but this allometric link is altered by the depth incubation treatment. (B) Relationship between the time taken to run 50 cm (log(seconds+1)) and the interaction between incubation temperature (°C) and the depth treatment, shown in residuals. The uncorrected version is provided in Figure S1D. (C) Time taken to self‐right (log(seconds+1)) is slower for hatchlings that emerged from sub‐clutches incubated in the shallow treatment. A version split by maternal ID (the random effect) is provided in Figure S1E.

Across all 10 clutches, we sampled 408 hatchlings (*n* = 198 from 20 deep sub‐clutches, *n* = 210 from 20 shallow sub‐clutches) for morphometrics and fitness tests. As expected, there was a positive correlation between hatchling SCL and mass (*F* = 281.7, *p* < 0.001), with heavier hatchlings per unit of length in the deep treatment (Mass × Treatment: *F* = 5.82, *p* = 0.016, Figure 2A, Table S2E for post hoc comparisons). Hatchling SCL also correlated with the interaction between sub‐clutch size and treatment (Sub‐clutch size × Treatment: *F* = 12.4, *p* < 0.001), whereby smaller sub‐clutch sizes were associated with increased hatchling SCL, with a steeper decline in the deep treatment (Figure S1C, Table S2E for post hoc comparisons).

We next measured the locomotion capacity of hatchlings, which is essential for dispersal and predator avoidance immediately upon emergence. Mean run time was correlated with an interaction between depth treatment and mean incubation temperature (*F* = 19.63, *p* < 0.0001, Figure 2B, Figure S1D for uncorrected version). With increasing temperatures, a sharp decline in run time was observed in shallow‐incubated hatchlings, in contrast to a slight increase in deep‐incubated hatchlings (Table S2F for post hoc comparisons). We further found that for a given sub‐clutch size, hatchlings from the deep sub‐clutches consistently outperformed hatchlings from shallow sub‐clutches, particularly in lower sub‐clutch sizes (Sub‐clutch size × Treatment: *F* = 4.284, *p* = 0.040, Table S2F for post hoc comparisons). There was no correlation between self‐righting time and mean incubation temperature, sub‐clutch size or hatchling size, which were all removed from the final model. Instead, the best predictor of self‐righting was the depth treatment alone, with hatchling emerging from the shallow sub‐clutches being slower at self‐righting (*F* = 11.489, *p* < 0.001, Figure 2C, Figure S1E for version split by maternal ID).

### 3.3 Global DNA Methylation Patterns

**FIGURE 3:**
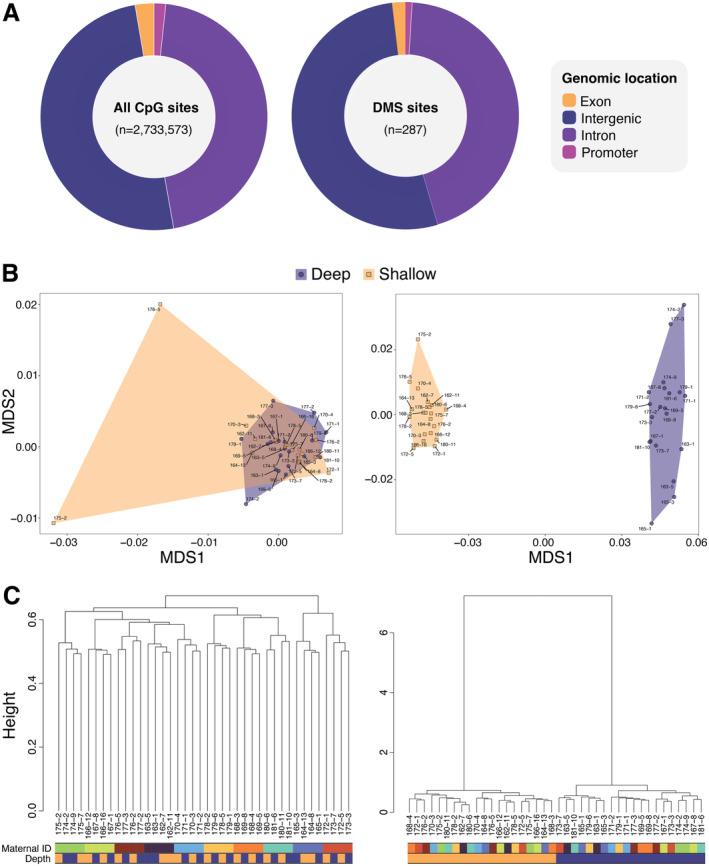
DNA methylation patterns of 40 hatchlings sequenced via WGBS. All plots on the left‐hand side show global methylation (*n* = 2,733,573 CpG sites) results, and all plots on the right‐hand side show results for DMS (*n* = 287 CpG sites). (A) Proportions of CpG sites across four genomic feature types. Left: Global CpG sites on gene promoters (1.67%), exons (2.68%), introns (45.55%) and intergenic space (50.1%). Right: DMS, on gene promoters (1.05%), exons (1.74%), introns (123%) and intergenic regions (54.4%). CpG sites were similarly distributed across genomic features between global and DMS categories. (B) NMDS plots with hatchlings coloured by treatment (shallow in orange, deep in blue). Left: Global methylation. Right: DMS. Clear clustering by incubation treatment can be seen in the DMS but not in the global methylation plot. (C) Hierarchical clustering dendrograms, with colour bars underneath the dendrogram representing each hatchling's maternal ID (top bar) and depth treatment (bottom bar, with shallow in orange, deep in blue). Left: Global methylation. Right: DMS. Maternal ID best explains clustering in at the global methylation level, whereas DMS clustered clearly by depth treatment.

For a subset of 40 hatchlings (*n* = 20 per depth treatment, *n* = 2 per sub‐clutch), we collected blood cell samples immediately after fitness tests for WGBS. This enabled us to investigate how global DNA methylation patterns are impacted by incubation conditions on a genome‐wide scale, using our dataset of 2,733,573 CpG sites that were covered in all individuals. These CpG sites were distributed across gene promoters (*n* = 45,627, 1.67%), exons (*n* = 73,374, 2.68%), introns (*n* = 1,245,062, 45.55%) and intergenic space (*n* = 1,369,510, 50.1%) throughout the genome (Figure 3A).

As expected, NMDS and hierarchical clustering of methylation values at 2,733,573 CpG sites showed that hatchlings do not form separate clusters by depth treatment on a whole genome level (Figure 3B,C, Figure S3A–D for accessory NMDS plots). NMDS results were robust even after the exclusion of two potential outliers that passed all quality controls (sample IDs: 175–2 and 176–5, Figure S3A–D for NMDS plots produced without these samples). The lack of grouping by depth treatment was confirmed by a PERMANOVA (*R* ^2^ = 0.024, *F* = 0.98, *p* = 0.80). Instead, we showed that maternal ID best explained the clustering of global methylation variance (*R* ^2^ = 0.25, *F* = 1.11, *p* = 0.001), which is also reflected in the hierarchical clustering analysis (Figure 3C). The lack of an important role for depth treatment in determining global methylation is further supported by our finding that the total count of methylated CpG sites per individual does not differ between hatchlings incubated at different depth treatments, in either the count of sites with non‐zero methylation (*F* = 0.041, *p* = 0.84, Figure S2A) or sites with > 70% methylation (*F* = 0.115, *p* = 0.74, Figure S2B).

### 3.4 DMS Identified Between Incubation Treatments

Although incubation treatment did not leave a detectable molecular signature at the whole methylome‐level, we identified 287 DMS between hatchlings from different depth treatments, with a mean methylation difference of 17.78 ± 2.26 (SD)% across all DMS (Figure S4). Methylation differences ranged from 26.49% hypermethylated (*n* = 109) in hatchling from the cooler, deep treatment, to 31.56% hyper‐methylated (*n* = 178) in hatchlings from the warmer, shallow treatment (i.e., equivalent to hypomethylated against hatchlings from the deep treatment, Figure S5). These DMS were located across gene promoters (*n* = 3, 1.05%), exons (*n* = 5, 1.74%), introns (*n* = 123, 42.9%) and intergenic regions (*n* = 156, 54.36%), which were similarly distributed across those genomic features compared to global CpG sites (χ^2^ = 0.597, *p* = 0.897, Figure 3A).

Hatchling methylation values at these 287 DMS generated clear clustering by depth treatments in the NMDS plot and hierarchical clustering analysis (Figure 3B,C, Figure S3A–D). A PERMANOVA confirmed that clustering at DMS is associated with the depth treatment (*R* ^2^ = 0.34, *F*_1,39_ = 19.75, *p* = 0.001) and not maternal ID (*R* ^2^ = 0.17, *F*_1,39_ = 1.112, *p* = 0.258), in contrast to results found at the global methylation level. Overall, methylation variance at these 287 DMS strongly discriminated between hatchlings that experienced the cooler, deep treatment and warmer, shallow treatment.

### 3.5 Genes Associated With DMS

From the 287 DMS, 148 sites were associated with a gene via overlap or proximity to the TSS (< 10 kb away) if it was intergenic (Table S3). Of these, 109 sites were hypermethylated in deep‐incubated hatchlings, versus 178 sites being hypermethylated in shallow‐incubated hatchlings (Figure S5). One gene had three DMS, five genes had two DMS and 135 genes had one DMS located on them. Given that differentially methylated CpG sites tend to co‐occur in clusters in the genome (Suzuki and Bird 2008), the small number of genes with multiple DMS in our dataset is likely due to our stringent filtering for sites covered in all individuals.

**FIGURE 4:**
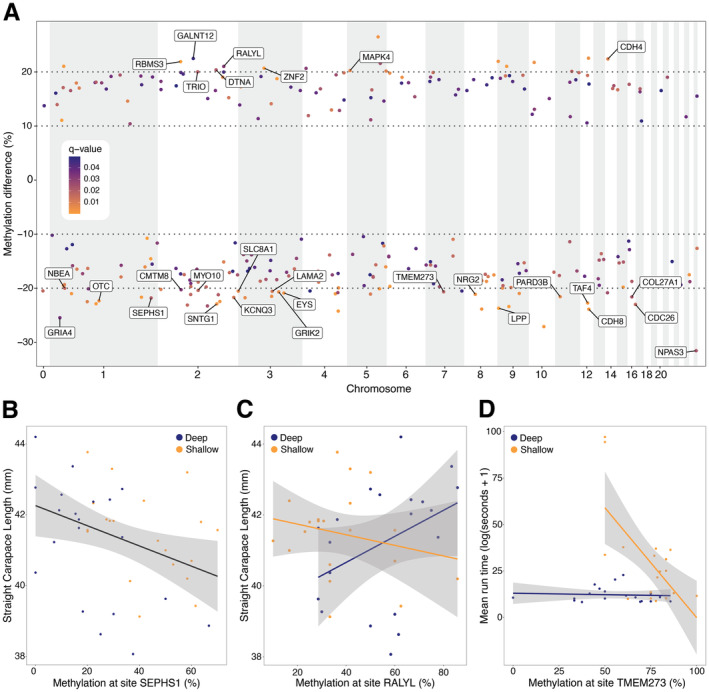
Methylation results in relation to DMS of interest. (A) Manhattan plot of the location of all DMS (*n* = 287, methylation difference > 10%, *q*‐value < 0.05) across the genome against methylation difference. A positive value indicates hypermethylation in deep‐incubated hatchlings, whereas a negative value indicates hypermethylation in shallow‐incubated hatchlings. Points are coloured by q‐value, and the 29 DMS of interest (methylation difference > 20%) are labelled. (B) Negative relationship between methylation (%) per hatchling at the DMS on the SEPHS1 gene with straight carapace length (SCL). (C) Interaction by treatment between methylation (%) per hatchling at the DMS on the RALYL gene against SCL. (D) Interaction by treatment at the DMS on the TMEM273 gene against mean run time (log(seconds+1)).

We further investigated the functions of genes associated with 29 DMS of interest that exhibited the highest mean methylation differences (> 20%) between hatchlings from different treatments (Figure 4A, Table S4). Of this subset, DMS on eight genes had higher methylation values in deep‐incubated hatchlings and DMS on 11 genes had higher methylation values in shallow, warm‐incubated hatchlings. These sites were spread across 10 chromosomes and mostly located on introns (*n* = 26). Non‐intronic DMS were more methylated in shallow‐incubated hatchling, with two found on exons (LAMA2, difference = 21.80%; TMEM273, difference = 20.66%), and one found in intergenic space < 10 kb from the nearest TSS (SEPHS1, difference = 21.80%). Interestingly, the LAMA2 (Laminin Subunit Alpha 2) gene encodes part of the laminin‐2 protein, which provides structural support and enables the transmission of force from muscle fibres to the extracellular matrix. In humans, mutations to LAMA2 can lead to congenital muscular dystrophy characterised by muscle weakness, respiratory problems and delayed motor development (El Kadiri et al. 2021; Tan et al. 2021). The TMEM273 (Transmembrane Protein 273) gene encodes a transmembrane protein embedded in the cell membrane. The exact function of the TMEM273 protein is not well understood, though it may play a role in craniofacial, muscle and skeletal development (Adams et al. 2024). Meanwhile, SEPHS1 (Selenophosphate Synthetase 1) was one of four enzyme‐encoding genes associated with the 29 DMS of interest. SEPHS1 is involved in regulation of cellular redox homeostasis to control the accumulation of reactive oxygen species, leading to growth retardation and even embryo death when dysregulated (Bang et al. 2022). The three other enzyme‐related genes are involved in different cellular processes: cellular signalling (MAPK4; Mitogen‐activated protein kinase 4; difference = 20.24%), nitrogen metabolism (OTC; Ornithine Transcarbamylase; difference = 22.34%) and protein glycosylation (GALNT12; Polypeptide N‐Acetylgalactosaminyltransferase 12; difference = 22.49%).

The most hypermethylated DMS in deep‐incubated hatchlings was located on the GALNT12 gene (difference = 22.49%). The widespread expression of this enzyme suggests an important role in protein glycosylation across the body. The most hypermethylated DMS in shallow‐incubated hatchlings was detected on the NPAS (Neuronal PAS domain protein 3) gene (difference = 31.56%). This neurodevelopmental gene influences the proliferation, differentiation and survival of neural progenitor cells, essential for brain formation during embryonic development (Kamm et al. 2013; Yang et al. 2016). The gene with the most detected DMS was RALYL (RNA Binding Protein Like), with two DMS more methylated in deep‐incubated hatchlings (mean difference = 20.50%) and one DMS more methylated in shallow‐incubated hatchlings (difference = 15.14%). This gene encodes a protein from a family of heterogeneous nuclear ribonucleoproteins with important roles in RNA processing, transport and metabolism. In humans, RALYL speculatively contributes to brain development and function, with links to neurodegenerative diseases like Alzheimer's and Parkinson's diseases (Zhang et al. 2020). DMS were also found on other neurodevelopmental genes, such as NRG2 (Pro‐neuregulin 2; difference = 21.13%) and NBEA (Neurobeachin; difference = 31.56%), which are both involved in synaptic regulation, as well as GRIK2 (Glutamate receptor Ionotropic Kainate 2; difference = 20.68%) and GRIA4 (Glutamate receptor 4; difference = 25.46%), both encoding glutamate receptors of the central nervous system.

### 3.6 Correlating Methylation Value at DMS of Interest Against Fitness‐Related Phenotypes

We further tested the relationship between hatchling fitness‐related traits (SCL, mass, mean run length, mean self‐righting time) and methylation value per individual at the 29 DMS of interest. After correcting for multiple testing, we found that SCL was correlated with methylation values at the DMS located on the SEPHS1 gene (*F* = 11.94, *p* = 0.002), with higher methylation associated with shorter SCL (Figure 4B). We also found that SCL was best explained by an interaction between depth treatment and DNA methylation at a DMS on the RALYL gene (*F* = 9.834, *p* = 0.004). At this site, higher methylation values were associated with longer SCL in hatchlings from the deep treatment, whereas the opposite was true for shallow‐incubated hatchlings (Figure 4C, Table S2J for post hoc comparisons). In relation to running ability, we found an interaction between depth treatment and DNA methylation at the DMS on the TMEM273 gene (*F* = 9.294, *p* = 0.005). Higher methylation values were associated with lower run times in hatchlings from shallow sub‐clutches, but methylation value did not change with run time in hatchlings from deep sub‐clutches (Figure 4D, Table S2K for post hoc comparisons). Interestingly, this reflects the same interaction found between run time and incubation temperature (Figure 2D).

### 3.7 Functional Enrichment of GO Terms

**FIGURE 5:**
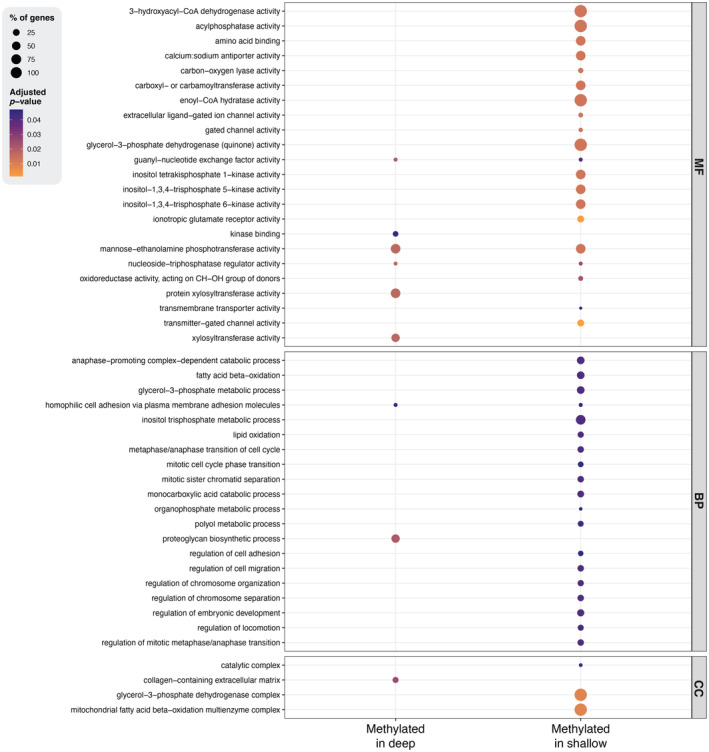
Functional enrichment of GO terms associated with genes with DMS in hatchlings from the incubation treatments. Sites with higher methylation values in hatchlings from the deep treatment are shown on the left, whereas sites with higher methylation values in hatchlings from the shallow treatment are shown on the right. The top panel shows GO terms related to molecular functions (MF), the middle panel shows GO terms related to biological processes (BP), and the bottom panel shows GO terms related to cellular functions (CF). Dot size represents the percentage of genes of a given term enriched in the dataset. The colour scale shows adjusted *p*‐values.

To investigate functions of identified DMS more broadly than at an individual gene level, we performed a GO term enrichment analysis for all gene‐associated DMS with available GO terms (*n* = 94). We identified 21 enrichments for biological processes, of which 19 were linked to hypermethylation in shallow‐incubated hatchlings and two in deep‐incubated hatchlings (Figure 5, Table S5). The two processes associated with methylation in deep‐incubated hatchlings were homophilic cell adhesion via plasma membrane adhesion molecules and proteoglycan biosynthetic processes. Homophilic adhesion is involved in cell–cell interactions and tissue organisation and was enriched in both treatments. Proteoglycan biosynthesis is important for the functioning of the extracellular matrix and was only enriched in the deep‐incubated hatchlings. Amongst the 19 biological processes enriched in shallow‐incubated hatchlings, seven were linked to cell‐cycle regulation, particularly during mitosis and the separation of sister chromatids. Four terms were associated with lipid and fatty acid metabolism. Triglycerides are stored in adipose tissue and mobilised as free fatty acids and glycerol when energy is needed. The breakdown of fatty acids to acetyl‐CoA in the mitochondria is important for producing ATP in tissues with high energy demands, like the heart and muscles.

Enrichment of molecular functions was also more prevalent in hatchlings from shallow treatments, with 20 terms versus six in their deep‐incubated counterparts. Of the 20 terms, these included five related to enzyme activities, five related to ion transport and gated channels and three related to inositol phosphate metabolism and signalling. Three out of the six terms enriched in deep‐incubated hatchlings were unique to the deep treatment, which related to enzyme activity and kinase binding. Finally, we detected one cellular process that was enriched in deep‐methylated hatchlings (collagen‐containing extracellular matrix), alongside three processes enriched in shallow‐incubated hatchlings, which are all essential in energy production and lipid metabolism (catalytic complex, glycerol‐3‐phosphate dehydrogenase complex and mitochondrial fatty acid beta‐oxidation multienzyme complex).

## 4 Discussion

As climate change intensifies, the proximal, thermal responses of individuals will be physiological and likely sublethal. Monitoring these effects in threatened species is essential, but reliable and high‐throughput molecular markers are needed. By experimentally manipulating incubation temperatures to simulate predicted future conditions, we found that warmer temperatures altered fitness‐related morphometric and locomotory traits, despite high and comparable hatching success rates between incubation treatments. Next, using whole‐genome bisulfite sequencing, we found 287 CpG sites that were consistently differentially methylated between hatchlings from the deep, cooler and shallow, warmer treatments. This demonstrates that incubation temperature leaves detectable signatures in the blood methylomes of loggerhead sea turtle hatchlings. Overall, our results indicate that even when sea turtles survive high incubation temperatures, they can still suffer negative fitness consequences in key physiological traits that impact dispersal ability and survival. Our study also brings evidence that DNA methylation can be used as potential biomarkers of early life thermal stress and their associated sublethal effects.

To evaluate the effects of temperature and incubation conditions on hatchlings, we used a split clutch design in an in situ hatchery. This experimental approach is valuable for assessing environmental impacts on species, independently of their genetic background and maternal effects. Whilst extensively used in laboratory settings (e.g., Eizaguirre et al. 2012; Kaufmann et al. 2014), such approaches are still infrequently applied in field conditions. Our findings reveal negative impacts of the thermal incubation regime on important fitness‐related phenotypes of hatchlings, in particular their length–mass relationship as well as their crawling and self‐righting capacity. These effects were captured with an average temperature difference of 0.83°C between depth treatments over 2 months of incubation, emphasising the vulnerability of this ectothermic species to global warming. Interestingly, we detected several context‐dependent fitness correlations. For instance, the positive mass–size relationship in hatchling was altered by depth treatment, being weaker in shallow‐incubated hatchlings, demonstrating the role of incubation environment in determining this allometric relationship (Parker and Begon 1986). Similarly, the negative correlation between hatchling size and sub‐clutch size also differed between depth treatments, being weaker in shallow‐incubated hatchlings. This could represent a case of context dependency, whereby the influence of egg number on nest microclimate (e.g., Reid, Monaghan, and Ruxton 2000) and energetic trade‐offs between offspring size and number in reptiles (e.g., Sinervo 1990; Ljungström et al. 2016) is further modified by the incubation environment. Despite longer run times observed in the shallow‐incubated hatchlings overall, running ability surprisingly improved with increasing temperature in shallow but not deep‐incubated hatchlings. This might be a by‐product of altered allometric relationships that are beneficial for this locomotory task at the measured temperature range. Overall, by employing a split‐clutch design in situ, we reveal context‐specific genotype‐by‐environment interactions linking hatchling fitness, maternal contribution (genetic and maternal effects) and incubation environment.

Our results align with previous suggestions of reduced fitness in loggerhead sea turtles under higher incubation temperatures (e.g., Reece et al. 2002; Booth 2017; Fleming et al. 2020). Crawling and self‐righting are part of the terrestrial dispersal phase immediately after emergence, which is followed by the swimming frenzy phase in the ocean (Scott et al. 2014). If temperature persistently hampers hatchling locomotion, it could impede their ability to disperse and colonise new habitats as climate change shifts nesting distributions (Perry et al. 2005; Kobayashi et al. 2018; Duffy, Gouhier, and Ganguly 2022). Reduced locomotion will also weaken their predator avoidance ability and secondarily their survival, which could diminish population recruitment rates (Schwanz et al. 2010; Hamilton et al. 2016). Furthermore, smaller hatchlings already exhibit reduced swimming capacity compared to larger counterparts (Mueller et al. 2019; Scott et al. 2014), so compounding effects on dispersal may be underestimated. If these impacts continue beyond the dispersal stage, carryover effects across life stages could also be underestimated. Of note, it is likely that different sexes have been induced in our experimental design, where eggs of a TSD species were incubated at different temperatures. Sex is unknown in this study as hatchling sacrifice or retention for laparoscopy for sexing was not conducted, but future work could test whether observed fitness differences are influenced by sex, which might be expected under the Charnov‐Bull model of TSD evolution (Charnov and Bull 1977).

In the context of sea turtles, hatchery experiments have often focused on management and reduction of incubation temperatures to mitigate lethal effects of increasing temperatures (e.g., Clarke et al. 2021; Esteban et al. 2018; Yao et al. 2022). Here, the experimental nests did not approach the lethal limits of eggs and embryos, as indicated by high, comparable hatching success rates between sub‐clutches incubated across both depth treatments. Instead, it helped reveal that hatching success rate may be a clutch‐specific trait independent of incubation conditions at sublethal thermal ranges, where small and large clutch sizes exhibited increased failures compared to intermediate clutch sizes. This relationship may represent the impact of clutch size on hatching success through altered nest microclimate (Reid, Monaghan, and Ruxton 2000) or maternal effects such as maternal body condition and investment strategy (e.g., Litzgus, Bolton, and Schulte‐Hostedde 2008; Wallace et al. 2007; Lockley et al. 2020; Fouda et al. 2023).

By pairing our findings of phenotypic fitness reductions with the functional annotation of differentially methylated genes, we bring physiological and molecular evidence for important sublethal effects. This study adds 287 DMS to growing evidence that DNA methylation carries the signatures of temperature exposure, even within sublethal ranges (Le Luyer et al. 2017; Sheldon et al. 2020; Bock et al. 2022). These may represent epigenetic regulation of gene expression, either directly in response to temperature or indirectly in downstream pathways. For example, we discovered DMS on genes with wide‐ranging roles in neurodevelopmental processes. This includes the NPAS3 gene, with the most differentiated DMS found overall, which coordinates neurogenesis and has been associated with neurodevelopmental disorders when dysregulated (Kamm et al. 2013; Yang et al. 2016). Such disruptions could impede hatchling fitness through negative impacts on traits such as motor control, sensory detection and cognition. Beyond specific genes, the breadth of affected pathways highlights the potential for pervasive developmental disruption from small temperature shifts, as seen in other vertebrates (Metzger and Schulte 2017; Anastasiadi et al. 2021). Disruption of these diverse functions could provide a mechanistic explanation for observed fitness reductions in warm‐incubated hatchlings. For instance, hypermethylated genes in hatchlings incubated at higher temperatures were enriched for lipid and fatty acid metabolism processes. Given that hatchlings undergo highly active behavioural stages upon emergence, disruption to such energy‐related processes may hinder active dispersal (Jones et al. 2007; Gatto et al. 2022). Enrichment of methylated genes relating to cytoskeletal and cell cycle regulation was also found in shallow‐incubated hatchlings, which could indicate alterations to development and cell proliferation during embryonic growth at higher incubation temperatures (Sunyer et al. 2009).

We uncovered direct links between variation in hatchling fitness and DNA methylation on three genes. From the perspective of finding biomarkers to monitor cryptic, sublethal effects, such sites are especially promising, as they could be used to predict fitness metrics via blood samples. Methylation of the SEPHS1 gene is correlated with hatchling size. As SEPHS1 is linked to embryonic growth retardation and reduced embryo size in mice (Bang et al. 2022), this relationship could provide a mechanistic insight into the pathways involved in altered growth rate at different incubation temperatures in sea turtles (e.g., Fleming et al. 2020). Sites in the RALYL and TMEM273 genes displayed methylation changes in different directions and magnitudes between incubation treatments, against size and run time, respectively. This is likely due to the complex interactions between different mechanisms that simultaneously mediate gene expression across different networks in a context‐dependent manner (Ambrosi, Manzo, and Baubec 2017). In loggerhead sea turtles, expression changes have been detected in heat shock (Tedeschi et al. 2015), TSD and life history trait pathways (Chow et al. 2021) in response to temperature. Our results therefore contribute additional pathways that may be regulated under thermal stress.

There is growing interest in quantifying DNA methylation patterns in sea turtles, with studies exploring global methylation in relation to adult scute patterning (Caracappa et al. 2016) and embryonic sex in loggerhead sea turtles (Venegas et al. 2016). Recently, reduced representation sequencing was applied to identify sites that differ with age (Mayne et al. 2022) and adult sex in green sea turtles (Mayne et al. 2023). Our study adds the first whole genome survey of differential methylation in sea turtles in the context of thermal stress. Taken together, the 287 DMS identified could act as potential epigenetic biomarkers to monitor sublethal effects of incubation temperatures. This is strengthened by the strict thresholds we applied, whereby CpG sites had to be identified as differentially methylated across all hatchlings. Similar signatures of incubation stress have been found in hatchery‐reared versus wild individuals of coho salmon, where methylation variation was associated with rearing environment and the capacity for successful migration (Le Luyer et al. 2017). It was further shown that those early‐life methylation marks persisted into germ line cells (Leitwein et al. 2021). If this is true for sea turtles, it further opens the possibility for biomarkers that reflect signatures of incubation conditions at later life stages. Other, future steps for biomarker development involve trialling targeted methods to reliably capture methylation signatures at DMS in a cheap and efficient manner.

Of note, the main clustering determinant of global methylation patterns was maternal ID, which likely represents the genetic background of the clutches, as well as maternal effects (e.g., Unterberger et al. 2009; Sable et al. 2015). This supports a growing body of research documenting the strong influence of underlying genetics on DNA methylation (Yang et al. 2010; Hawe et al. 2022). For example, Sepers et al. (2023) showed that genetic background was a better predictor of DNA methylation than rearing environment in a wild great tit population. Meanwhile, genetic sequence explained variation in DNA methylation across 580 animal species, with the density of CpG sites and islands thought to be particularly important (Klughammer et al. 2023). Haghani et al. (2023) also showed robust phyloepigenetic‐phylogenetic congruence across 348 mammalian species. As molecular markers for monitoring responses to climate change are being developed, combining genetic and epigenetic data to disentangle the effects of environment versus genetics on methylation patterns will provide crucial insights.

Beyond sea turtles, the framework we introduce here—leveraging split‐clutch experimental designs to identify epigenetic biomarkers associated with climate‐mediated stressors in a natural setting—could be widely applicable to conservation biology questions. Although many laboratory and captive studies have documented thermal effects on epigenetic patterns, demonstrating these relationships in natural contexts remains challenging. Manipulative studies in the field are thus invaluable, yet still rare in ecological epigenetics, especially in non‐model species (Chapelle and Silvestre 2022). Our findings showcase their utility for strengthening claims of association between epigenetic variation and environmental stressors. Expanding the epigenetic toolkit to other wild populations could similarly reveal subtle impacts of climate threats that evade detection by traditional survival‐oriented metrics. Well‐designed field studies in non‐model systems will provide the most realistic insights into the interplay between phenotypic fitness, epigenetic regulation and population resilience across ecological contexts in the wild, which is a priority for advancing predictive, mechanistic understanding in conservation epigenetics.

## 5 Conclusions

We show that beyond mortality, warming could have pervasive yet cryptic sublethal impacts on sea turtles. Even if lethal thresholds are not crossed, subtler fitness reductions could still accelerate population declines, especially if impacts in early life are carried over to later life stages. Such impacts are detectable through DNA methylation status at identified genomic sites. As these molecular markers carry signatures of thermal stress induced by the incubation environment, they could provide a means to monitor early warning signals of sublethal effects at high temperatures, complementing demographic perspectives. Our study also highlights the complex interplay between genetics and environment that will shape population trajectories and therefore species persistence in a changing world. Much work remains to unravel these dynamics in non‐model species, yet emerging conservation epigenetic approaches offer promising tools to meet the challenges ahead.

## Conflicts of Interest

Alice Balard is an Editorial Board member of Evolutionary Applications and a co‐author of this article. To minimise bias, they were excluded from all editorial decision‐making related to the acceptance of this article for publication.

## Permits

All sample collection and experiments adhered to national legislation and were approved by the Direção Nacional do Ambiente de Cabo Verde (authorisation: 037/DNA/2021).

## Supporting information


**Figure S1.** Relationships of mean incubation temperature with incubation duration and clutch size.
**Figure S2.** Counts of methylated CpG sites per individual.
**Figure S3.** NMDS plots showing 1–3 MDS dimensions.
**Figure S4.** Volcano plot of the 287 DMS identified between incubation treatments.
**Text S1.** Extended methods for SNP calling pipeline.
**Table S1.** WGBS statistics per hatchling.

**Table S2.** All regression model formulations and outputs.

**Table S3.** Functional annotation of all 148 gene‐associated DMS.

**Table S4.** Names and functional annotation of the 29 DMS of interest.

**Table S5.** GO enrichment results table.

## Data Availability

All sequencing data are available under ENA study accession number PRJEB75968. All coding scripts are available at: https://github.com/eugeniecyen/Article_CarCar_ThermalSublethalMeth.
