# Supplementary material for: DNA Methylation Carries Signatures of Sublethal Effects Under Thermal Stress in Loggerhead Sea Turtles
Source: Evol Appl. 2024 Sep 15;17(9):e70013. doi: 10.1111/eva.70013 (PMC11403127; doi:10.1111/eva.70013)
Supplement: Supplementary file 1 — Figure S1. Relationships of mean incubation temperature with incubation duration and clutch size. Figure S2. Counts of methylated CpG sites per individual. Figure S3. NMDS plots showing 1–3 MDS dimensions. Figure S4. Volcano plot of the 287 DMS identified between incubation treatments. Text S1. Extended methods for SNP calling pipeline. Table S1. WGBS statistics per hatchling. [file EVA-17-e70013-s005.docx]

**Supplementary Material**

**DNA methylation carries signatures of sublethal effects under thermal stress in sea turtles**

**Authors**

Eugenie C. Yen^1*^, James D. Gilbert^1^, Alice Balard^1^, Inês O. Afonso^1^, Kirsten Fairweather ^2^, Débora Newlands^2^, Artur Lopes^2^, Sandra M. Correia^3^, Albert Taxonera ^2^, Christophe Eizaguirre ^1^

**Affiliations**

^1^ Queen Mary University of London. School of Biological and Behavioural Sciences, London, UK
^2^ Project Biodiversity, Mercado Municipal, local 22 Santa Maria, Ilha do Sal, Cabo Verde
^3^ Instituto do Mar (IMar), Cova dÍnglesa, CP132 Mindelo, Ilha do São Vicente, Cabo Verde

* **Corresponding Author**: Eugenie C. Yen. School of Biological and Behavioural Sciences, Queen Mary University of London, London, E1 4DQ, UK. Email: [e.yen@qmul.ac.uk](mailto:e.yen@qmul.ac.uk).

**Supplementary Figure 1.** Additional relationships detected between phenotypes at the clutch- and hatchling-level. Deep sub-clutches are coloured in blue and shallow sub-clutches in orange. **(A)** Correlation between incubation duration (days) and mean incubation temperature (^o^C) per sub-clutch. The total incubation duration is shorter in sub-clutches incubated at higher temperatures (F_1,12_=17.33, p<0.001). **(B)** The effect of metabolic heat, as shown by the positive correlation between incubation temperature and nest size. Note, the interaction between the depth treatment and the clutch size is uncorrelated with metabolic heat, as expected in a split-clutch experimental design (Treatment x Sub-clutch size, F_1,12_= 1.3166, p=0.273). (**C)** The interaction between clutch size and hatchling straight carapace length, SCL (Clutch size x Treatment: F=4.462, p=0.035). SCL is negatively correlated with clutch size, and this link is altered by depth treatment. (**D)** Mean run time per hatchling is correlated with an interaction between depth treatment and mean incubation temperature residuals (F=19.63, p<0.0001). **(E)** Self-righting time per hatchling, split by treatment and the random effect maternal ID. Shallow-incubated hatchlings are slower at self-righting than their deep-incubated counterparts (F= 11.489, p<0.001).


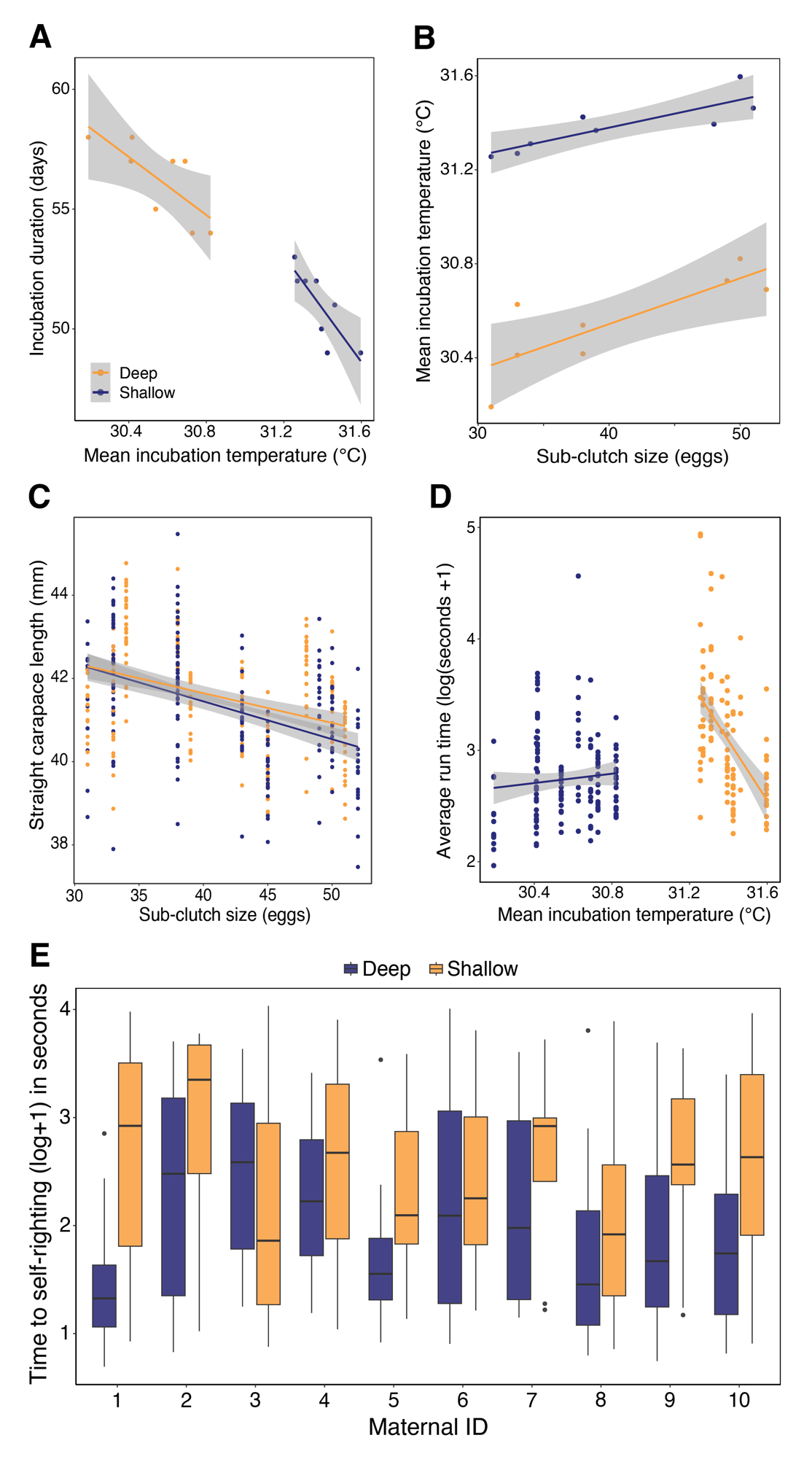


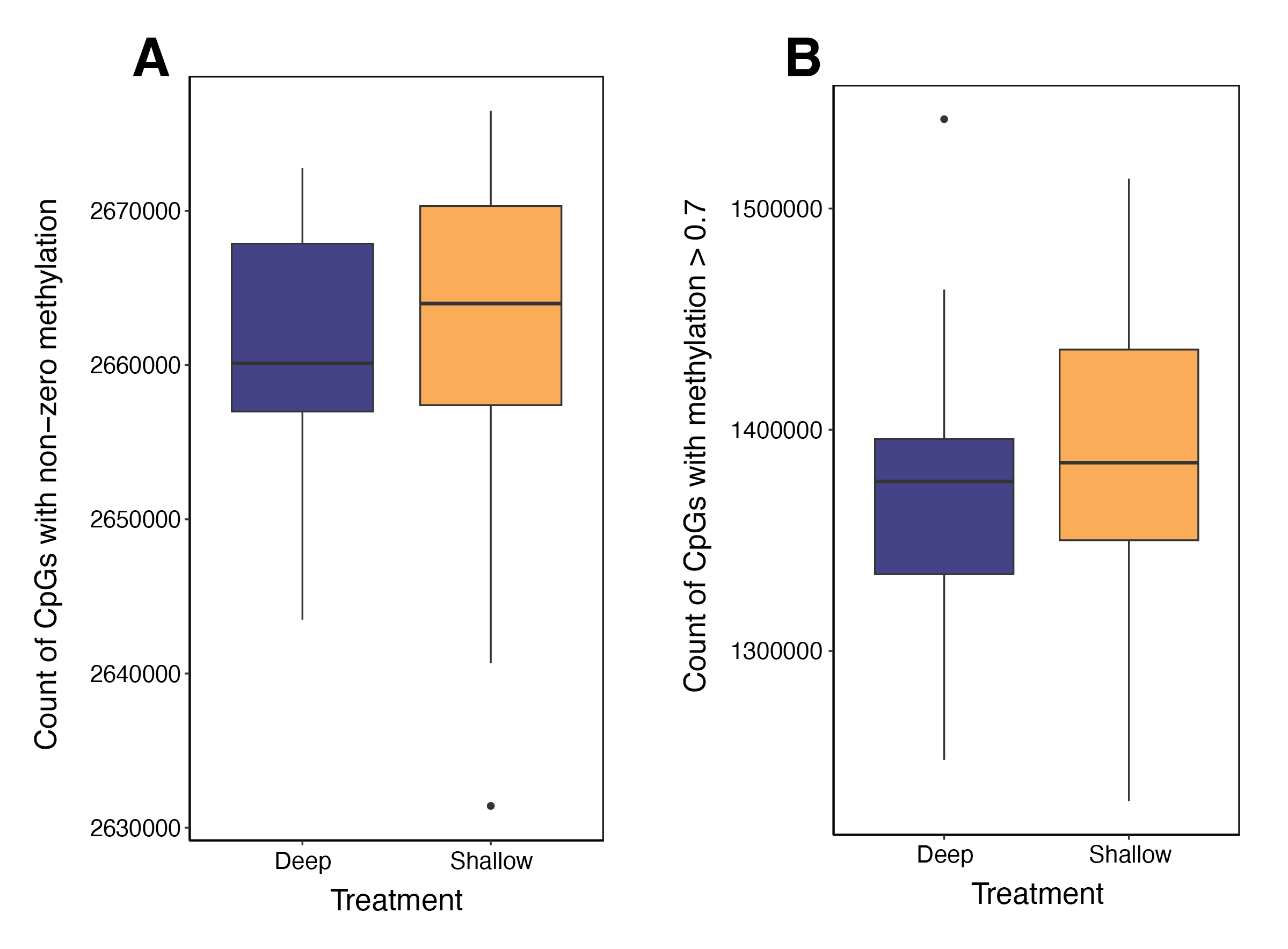
**Supplementary Figure 2.** Counts of methylated CpG sites per individual. Deep-incubated hatchlings are in blue and shallow-incubated hatchlings are in orange. **(A)** Count of CpG sites with non-zero (>0%) methylation values per individual. **(B)** Count of CpG sites with a methylation proportion above 0.7 (>70%) per individual.


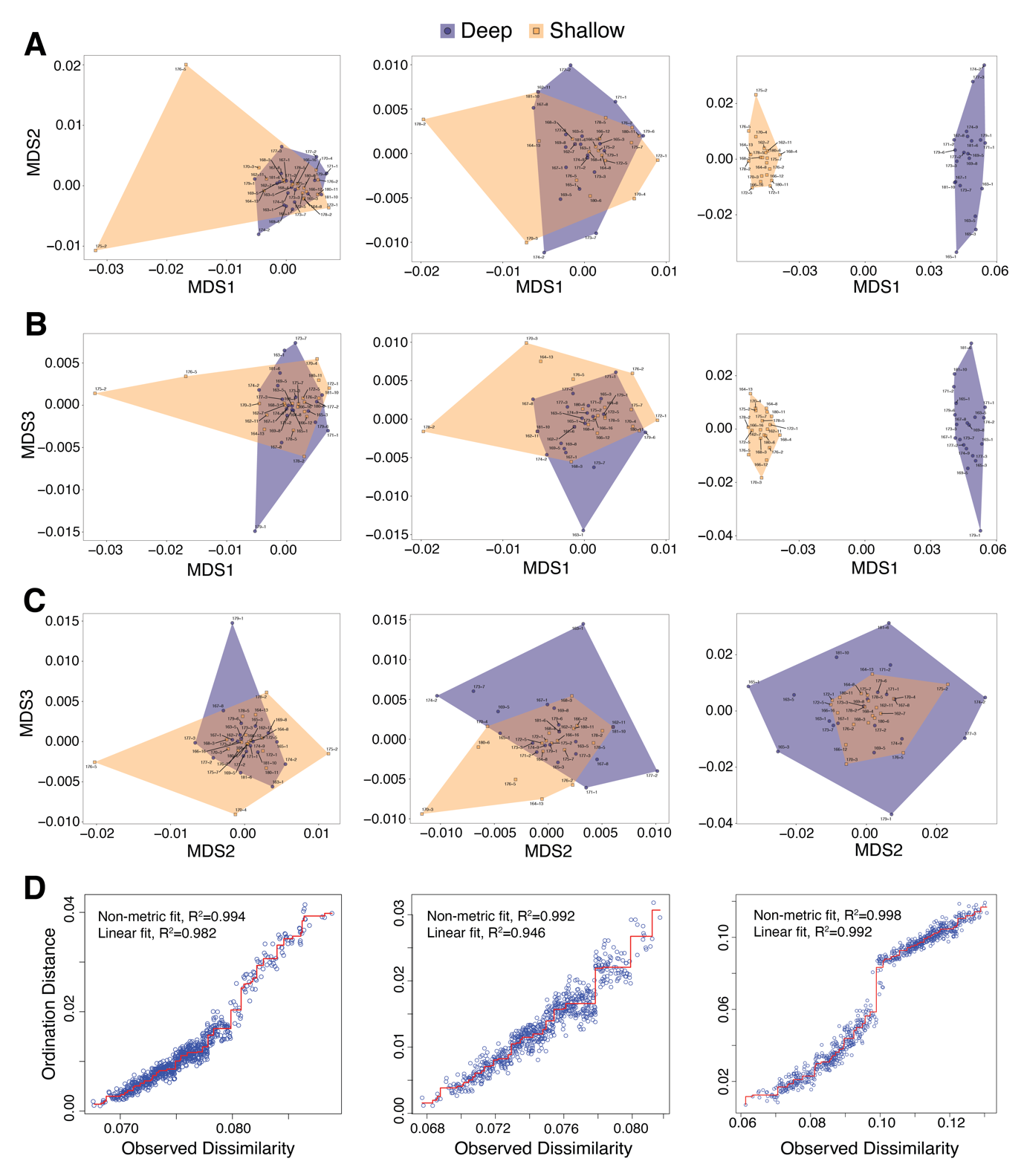
**Supplementary Figure 3.** NMDS plots with additional dimensions. All plots in the left column show global methylation (n=2,733,573 CpG sites) results. All plots in the middle column show global methylation (n=2,733,573 CpG sites) with two potential outlier individuals excluded (IDs: 175-2 and 176-5). All plots in the right column show DMS results (n=287 CpG sites). Plots of dimensions **(A)** MDS1 versus MDS2, **(B)** MDS1 versus MDS3, **(C)** MDS2 versus MDS3, and **(D)** Stress plots of goodness of fit for NMDS.

**Supplementary Figure 4.** Volcano plot of the 287 DMS identified between hatchling from the different incubation treatments. DMS were selected with the threshold of having over 10% methylation difference between hatchling from the deep and shallow treatments, and a q-value less than 0.05. For each DMS, its methylation difference is plotted against the –log_10_(q-value). A positive methylation value indicates hyper-methylation in deep-incubated hatchlings and a negative value indicates hyper-methylation in shallow-incubated hatchlings.

**Supplementary Text 1.** Extended methods for SNP calling pipeline. To obtain a list of C-to-T SNPs to remove from the CpG dataset, the Revelio algorithm was used to mask bases generated by the bisulfite conversion process that may be incorrectly interpreted as SNPs (Nunn et al. 2022), then the GATK pipeline v.4.2.6.1 (McKenna et al. 2010) was used to call SNPs. Read group information was added to the masked, aligned reads with samtools v.1.10 (Li et al. 2010), followed by SNP calling using the GATK pipeline v.4.2.6.1 **(**McKenna et al. 2010). SNPs were called per sample with HaplotypeCaller. Joint genotyping was performed with GenotypeGVCFs across samples in the deep and shallow treatments separately. This was done to minimise the interference of true differentially methylated sites between depth treatments when calling C-to-T SNPs. This is because siblings in each treatment should have the same genetic background, but have different methylation patterns induced by the incubation condition experienced. Several filtering steps were performed next to give the final SNP dataset. We removed SNPs with (1) a quality by depth less than 2 and mapping quality less than 35 with GATK SelectVariants, (2) coverage in the 99.9^th^ percentile with vcftools v0.1.16 (Danecek et al. 2011), and (3) coverage less than 7 and within 10 bp of an indel with bcftools v1.16 (Danecek et al. 2021). Linkage disequilibrium pruning (r^2^<0.9 in 10 kb windows) was then applied using bcftools. Finally, SNPs with a minor allele frequency less than 0.05 were removed to produce the final SNP dataset for each treatment.

**Supplementary Table 1.** WGBS statistics per hatchling. For the subset of hatchlings that were selected for sequencing (n=40 total; n=20 per treatment; n=4 per 10 clutches, n=2 per 20 sub-clutches).

| Hatchling ID | Maternal ID | Treatment | Total raw read pairs | Mean mapping efficiency (%) | Bisulfite conversion efficiency (%) | Total CpGs after de-stranding | Mean CpG coverage after de-stranding |
| --- | --- | --- | --- | --- | --- | --- | --- |
| 162-7 | SLL063 | Shallow | 132125697 | 79.0 | 99.994 | 25420314 | 8.93 |
| 162-11 | SLL063 | Shallow | 138252128 | 80.7 | 99.995 | 24187368 | 7.77 |
| 163-1 | SLL063 | Deep | 132157135 | 74.3 | 99.994 | 25555409 | 9.00 |
| 163-5 | SLL063 | Deep | 138215181 | 80.8 | 99.993 | 23339675 | 7.48 |
| 164-8 | SLL065 | Shallow | 138154759 | 83.3 | 99.995 | 25551989 | 9.75 |
| 164-13 | SLL065 | Shallow | 132236373 | 81.7 | 99.994 | 24235846 | 7.83 |
| 165-1 | SLL065 | Deep | 132286877 | 79.5 | 99.994 | 25522943 | 9.40 |
| 165-3 | SLL065 | Deep | 124309977 | 78.5 | 99.993 | 22879483 | 6.60 |
| 166-12 | SLL146 | Shallow | 132168259 | 78.3 | 99.994 | 25550303 | 9.57 |
| 166-16 | SLL146 | Shallow | 138244303 | 73.6 | 99.993 | 23713831 | 7.16 |
| 167-1 | SLL146 | Deep | 138205348 | 68.2 | 99.992 | 23528252 | 6.68 |
| 167-8 | SLL146 | Deep | 132250210 | 78.2 | 99.994 | 25513139 | 9.06 |
| 168-3 | SLL176 | Shallow | 132310832 | 77.4 | 99.994 | 25489077 | 9.04 |
| 168-4 | SLL176 | Shallow | 139288016 | 82.2 | 99.995 | 25539371 | 10.08 |
| 169-5 | SLL176 | Deep | 138547819 | 80.5 | 99.995 | 25551370 | 9.90 |
| 169-8 | SLL176 | Deep | 132354672 | 75.8 | 99.993 | 25318231 | 8.27 |
| 170-3 | SLL142 | Shallow | 138564032 | 78.8 | 99.995 | 25532667 | 9.76 |
| 170-4 | SLL142 | Shallow | 132251390 | 72.8 | 99.989 | 25434866 | 8.54 |
| 171-1 | SLL142 | Deep | 132277642 | 74.1 | 99.990 | 25426705 | 8.58 |
| 171-2 | SLL142 | Deep | 139003709 | 82.0 | 99.995 | 25511930 | 9.82 |
| 172-1 | SLL188 | Shallow | 132302522 | 75.3 | 99.991 | 25428030 | 8.60 |
| 172-5 | SLL188 | Shallow | 138373670 | 77.1 | 99.992 | 24004894 | 7.42 |
| 173-3 | SLL188 | Deep | 128768337 | 83.4 | 99.994 | 23728787 | 7.37 |
| 173-7 | SLL188 | Deep | 132340278 | 77.9 | 99.992 | 25500965 | 9.10 |
| 174-2 | SLL144 | Deep | 132334347 | 79.7 | 99.991 | 25438012 | 8.91 |
| 174-9 | SLL144 | Deep | 138210202 | 81.8 | 99.994 | 23603725 | 7.58 |
| 175-2 | SLL144 | Shallow | 132255878 | 75.9 | 99.991 | 25487153 | 8.90 |
| 175-7 | SLL144 | Shallow | 138178365 | 81.4 | 99.995 | 24426063 | 7.71 |
| 176-2 | SLL189 | Shallow | 138698534 | 79.5 | 99.995 | 25541878 | 9.68 |
| 176-5 | SLL189 | Shallow | 132235631 | 78.9 | 99.991 | 25465148 | 8.98 |
| 177-2 | SLL189 | Deep | 128738663 | 75.5 | 99.991 | 25391429 | 8.37 |
| 177-3 | SLL189 | Deep | 138622777 | 82.1 | 99.995 | 25514569 | 9.67 |
| 178-2 | SLL171 | Shallow | 132326833 | 75.3 | 99.991 | 25393011 | 8.48 |
| 178-5 | SLL171 | Shallow | 138221719 | 82.8 | 99.994 | 24782292 | 8.17 |
| 179-1 | SLL171 | Deep | 132307648 | 76.3 | 99.991 | 25488501 | 8.85 |
| 179-6 | SLL171 | Deep | 136149240 | 77.8 | 99.995 | 24299715 | 7.51 |
| 180-6 | SLL143 | Shallow | 132248782 | 78.0 | 99.991 | 25469284 | 8.92 |
| 180-11 | SLL143 | Shallow | 138139674 | 79.9 | 99.995 | 23336240 | 7.33 |
| 181-6 | SLL143 | Deep | 132307312 | 76.7 | 99.992 | 25404232 | 8.58 |
| 181-10 | SLL143 | Deep | 133734003 | 85.1 | 99.996 | 25396507 | 8.96 |

**Supplementary Table 2.** All regression model formulations and outputs (Excel)

**Supplementary Table 3.** Functional annotation of all 148 gene-associated DMS (Excel)

**Supplementary Table 4.** Names and functional annotation of the 29 DMS of interest (Excel)

**Supplementary Table 5.** GO enrichment results table (Excel)
